# Editorial: Precision Public Health

**DOI:** 10.3389/fpubh.2018.00121

**Published:** 2018-04-30

**Authors:** Tarun Stephen Weeramanthri, Hugh J. S. Dawkins, Gareth Baynam, Matthew Bellgard, Ori Gudes, James Bernard Semmens

**Affiliations:** ^1^Public and Aboriginal Health Division, Western Australian Department of Health, Government of Western Australia, Perth, WA, Australia; ^2^Genetic Services of Western Australia, Subiaco, WA, Australia; ^3^eResearch Directorate, Queensland University of Technology, Brisbane, QLD, Australia; ^4^University of New South Wales, Sydney, NSW, Australia; ^5^Curtin University, Perth, WA, Australia

**Keywords:** technology, data, GIS, equity, ethics, omics, prevention, policy

**Editorial on the Research Topic**

**Precision Public Health**

## Introduction—Old and New

Traditional public health practice has had a central reliance on data, and the core discipline of epidemiology, in order to inform health policy and priority setting, drive health improvement across whole populations, and target disadvantaged populations. Core public health activities include risk factor and disease surveillance, screening, development of interventions, assurance, and evaluation. Since the 1970s, New Public Health has also emphasized community engagement, health promotion, partnerships, and advocacy.

In the last 20 years, and particularly with the sequencing of the human genome and advances in other “-omics,” informatics and a range of technologies, new possibilities have opened up for a much more finely delineated view of the “time-person-place” triad that underpins epidemiology, and the balancing of genetic, biological, environmental, and social determinants of disease.

This may lead, we argue in this article, to new preventive and treatment options and the next paradigm shift in public health, namely toward “Precision Public Health” or PPH. However, we also caution against a blind optimism about what technology can achieve on its own, and argue for a solid grounding of PPH on the old verities of public health, namely whole population health improvement and equity.

## Use of the Term “Precision Public Health”

In 2013, building on our experience in the Health Department of Western Australia with genomics, spatial technology in health, and data linkage, and our extensive “policy-practice-academic” partnerships in all three areas, we proposed use of the term “Precision Public Health” to complement the parallel developments in medicine, such as Personalized Medicine and Precision Medicine, a term used in a 2011 US National Academy of Sciences Report, and then the subject of a major US research initiative in 2015, focused on cancer and other diseases (1).

Reservations about the individual and clinical focus of Precision Medicine, its silence on social determinants, and its capacity to improve population health were expressed by Bayer and Galea (2). The new concept of PPH was introduced into the academic literature by Khoury,^1^ who called for a modernization of surveillance, epidemiology, and information systems, as well as targeted interventions and a population health perspective (3). Most recently, Khoury has emphasized the historic continuity of PPH to work on public health genomics over recent decades, while acknowledging that PPH encompasses more than genomics (4).

The first meeting to use the “PPH” term was the Precision Public Health Summit held in San Francisco in June 2016.^2^ Though most of the participants were from the US, the meeting had a global health focus, and focused on data integration and sharing, new partnerships, community engagement, and social justice for better public health outcomes. A subsequent article from Bill and Melinda Gates Foundation authors presented a “back to basics” view of PPH suitable to the developing world: use of data with greater geographic precision to improve disease surveillance; better birth and death registration; building of laboratory capacity; and training in epidemiology (5).

## Definition of “Precision Public Health”

Though a universal definition of PPH has not been adopted, a number of complementary definitions have been proposed.

In the introduction to this Frontiers Research Topic (RT), we proposed the following definition of “precision public health”: “the application and combination of new and existing technologies, which more precisely describe and analyse individuals and their environment over the life course, to tailor preventive interventions for at-risk groups and improve the overall health of the population.”

The Precision Public Health Summit had a breakout group session on “Building a Working Definition of PPH,”^3^ where divisions emerged between clinicians and academics on one side, and public health practitioners on the other, on whether the goals of PPH were already encompassed under Precision Medicine, and whether an alternative hybrid term such as Precision Health was preferable. There was a clear perception that the PPH term carried an implied criticism of Precision Medicine, the fairness of which was debated.

Khoury has described “precision in the context of public health” as “improving the ability to prevent disease, promote health and reduce health disparities in populations” through the application of technology and the development of targeted programs and health policy (paraphrased) (see text footnote 1).

In this Frontiers RT, Dolley has described PPH as “an emerging practice to more granularly predict and understand public health risks and customize treatments for more specific and homogenous sub-populations, often using new data, technologies and methods.”

Baynam et al. has added a descriptor of PPH as a “new field driven by technological advances that enable more precise descriptions and analyses of individuals and population groups, with a view to improving the overall health of populations.”

## Key Questions

In this RT, we sought articles to kick-start this new concept by posing the following questions.

- What are the new “precision” technologies, and how might they affect existing public health policy and practice, and in which areas (e.g., wellness, illness, or disease states; if disease, communicable diseases or chronic diseases)?
- Will these new technologies be able to strengthen preventive strategies, improve access to health care, or reach currently neglected or disadvantaged populations?
- What new and old technologies need to be combined and/or integrated to radically advance public health policy and practice, and lead to improved quality and quantity of life?
- What can we learn from the history and ethics of public health that will allow us to creatively and purposively take advantage of new technologies, many of which are developed in the private sector?
- What are the downsides of the new technologies and how can these be mitigated (e.g., through education or appropriate policy, risk management, systems design, research, or regulatory frameworks)?

## RT Articles—Broad Categories

The 18 papers in the RT addressed in main the first three questions, as well as the last question, and can be grouped into the following broad and non-exclusive categories:

Genomics, newborn screening, phenomics, or other “omics” (Molster et al., Newnham et al., Baynam et al., Jansen et al.).

Spatial or GIS (Campbell and Ballas, Weeramanthri and Woodgate).

Data, analytics, and informatics (Brown et al., Lwin et al., Mann et al., Spilsbury et al., Gunnell et al., Xiao et al., Bellgard et al., Troeung et al., Preen et al., Dolley).

Case studies in infectious diseases (Inglis and Urosevic, Newnham et al.).

Case studies in cancer prevention, screening, and survival (Gunnell et al., Girschik et al., Troeung et al., Preen et al.).

Population vulnerability, equity, and targeted public health policy (Campbell and Ballas, Weeramanthri and Woodgate, Molster et al., Xiao et al., Newnham et al., Girschik et al., Jansen et al., Troeung et al.).

Ethics and privacy (Brown et al., Molster et al., Jansen et al.).

Surveillance and screening (Lwin et al., Inglis and Urosevic, Molster et al., Jansen et al., Troeung et al., Preen et al.).

Social media, mobiles, community participation, and crowdsourcing (Lwin et al., Girschik et al.).

## RT Articles—Specific Points

Newborn screening can be viewed as an archetypal PPH technology. Despite being introduced more than 50 years ago, Jansen et al. demonstrate there are many unanswered questions around evidence, affordability, policy, and the introduction of new tests as technology improves. Molster et al. show that consideration of preconception carrier screening needs careful balancing of potential harms against benefits.

Girschik et al. synthesize data, academic literature, and expert opinion into an explicit and precise process for setting cancer prevention priorities.

Lwin et al. show us how to apply new mobile technologies and crowdsourcing, to produce real-time surveillance data for influenza tracking.

Campbell and Ballas and Xiao et al. use complex spatial and other analytic methods to unlock administrative datasets to identify inequity and drive progressive policy.

Gunnell et al. show the value of linking administrative data to well-designed, longitudinal cohort studies, to derive precise measures of physical activity and mortality in cancer survivors.

Preen et al. and Troeung et al. examine colonoscopy data from administrative datasets to predict risk of colon cancer and target policy to particular age groups.

Inglis and Urosevic look at diagnostic and surveillance challenges of antimicrobial resistance in detail, and remind us of the need for validation of tools and tests, and the steps and pitfalls on the route from cell to bench to person to population.

Dolley and Mann et al. test the claims of “Big Data” enthusiasts, and offer alternatives.

The ethical implications of the new precision technologies for consent and privacy are addressed by Brown et al. in their article on data linkage.

Two papers test the value of PPH as a policy framework. Newnham et al. comprehensively examine the biological and social factors behind preterm birth, including evidence-based research in various “-omics” fields, so as to construct multilevel preventive policy. Baynam et al. sees 3-D facial analysis as a “prototypical precision public health tool” and show how phenotype complements genotype, and links to a traditional public health policy wheel.

Weeramanthri and Woodgate outline a set of recommendations to improve uptake and use of spatial data in the health sector, which could be applied to precision technologies in general. Their recommendations include communication of strong case studies, linkage of spatial data to patient pathways, formal cost-effectiveness analysis of the value added by technology, and training, capacity, and new stakeholder partnerships.

## Conclusion and Future Steps

Precision public health is a rapidly evolving field.

Any notion of precision must begin with an attention to precise and unambiguous language, which not only underpins definitional, measurement, and classification issues but also aids clear communication with the public and professional groups.

When we look at our original RT proposal, and compare the definition of PPH offered there, to the material in the papers that were submitted and accepted, it is clear that “data and informatics” needs to be front and central in any future consensus definition. It is the combination of data-related skills and technologies (e.g., in epidemiology, data linkage, informatics, and communications) and the ability to aggregate, analyze, visualize, and make available high quality data, larger or linked, in closer to real time, that is at the heart of PPH, much like epidemiology is at the heart of traditional public health.

Another challenge is to build on the work presented in this RT, which mainly comes from countries with developed economies (Australia, US, UK, Singapore), and explore how the concept can be applied in all countries, with varying levels of resources and health investment, struggling to provide universal health coverage.

To this end, the RT editors and others are organizing a Precision Public Health Asia Symposium^4^ to be held in October 2018, to further work on a consensus definition, to explore in more detail the ethical and social implications of the concept, and as a launchpad for further collaboration in the region.

This group of RT articles specifically reinforces the importance of embedding old and new technologies within explicit policy frameworks, whether traditional policy cycles or newer frameworks derived from systems biology or complexity theory (Inglis and Urosevic, Bellgard et al.). Such planning is central to operationalizing PPH, which sits at the nexus of precision medicine and public health, moving us from an “*n* of 1” (precision medicine) to an “*n* of many” (precision public health). It is a fundamental choice—new technologies leading by chance to more precise diagnoses and treatments for some fortunate individuals, or planning for and designing a system that offers those same benefits across the population and with a shorter lag time to those most in need.

## Author Contributions

TW drafted the Editorial, and all other authors revised and approved the final version.

## Conflict of Interest Statement

The authors declare that the research was conducted in the absence of any commercial or financial relationships that could be construed as a potential conflict of interest.

## Footnotes

^1^Khoury M. CDC Blog post March 2, 2015 titled “Precision public health and precision medicine: two peas in a pod.” Available from: https://blogs.cdc.gov/genomics/2015/03/02/precision-public/ (Accessed: April 18, 2018).

^2^https://precisionmedicine.ucsf.edu/programs/precision-population-health/summit (Accessed: April 18, 2018).

^3^https://tinyurl.com/yddwgsnq (Accessed: April 18, 2018).

^4^www.pph2018.com (Accessed: April 18, 2018).
